# Surprisingly Effective Priming of CD8^+^ T Cells by Heat-Inactivated Vaccinia Virus Virions

**DOI:** 10.1128/JVI.01486-20

**Published:** 2020-09-29

**Authors:** Sarah Croft, Yik Chun Wong, Stewart A. Smith, Inge E. A. Flesch, David C. Tscharke

**Affiliations:** a John Curtin School of Medical Research, The Australian National University, Canberra, Australia; University of Illinois at Urbana Champaign

**Keywords:** CD8^+^ T cells, T cells, antigen processing, vaccines, vaccinia virus

## Abstract

Robust priming of CD8^+^ T cells by viruses is considered to require infection and *de novo* expression of viral antigens. A corollary of this is that inactivated viruses are thought of as being inevitably poor vaccines for eliciting these responses. In contrast to this dogma, we found that some antigens present in vaccinia virus (VACV) virions prime strong CD8^+^ T cell responses when the virus was rendered noninfectious by heat. More surprisingly, in some cases these responses were similar in magnitude to those primed by infectious virus administered at an equivalent dose. Next, we tested whether this was a special property of particular antigens and their epitopes and found that foreign epitopes tagged onto three different VACV virion proteins were able to elicit CD8^+^ T cell responses irrespective of whether the virus was viable or heat killed. Further, the polyfunctionality and cytotoxic ability of the CD8^+^ T cells primed by these VACVs was equivalent irrespective of whether they were administered to mice as inactivated or live viruses. Finally, we used these VACVs in prime-boost combinations of inactivated and live virus and found that priming with dead virus before a live booster was the most immunogenic regime. We conclude that VACV virions can be efficient vectors for targeting antigens to dendritic cells for effective priming of CD8^+^ T cells, even when rendered noninfectious and speculate that this might also be the case for other viruses.

**IMPORTANCE** The design of viral vectored vaccines is often considered to require a trade-off between efficacy and safety. This is especially the case for vaccines that aim to induce killer (CD8^+^) T cells, where there is a well-established dogma that links infection in vaccinated individuals with effective induction of immunity. However, we found that some proteins of vaccinia virus generate strong CD8^+^ T cell responses even when the virus preparation was inactivated by heat prior to administration as a vaccine. We took advantage of this finding by engineering a new vaccine vector virus that could be used as an inactivated vaccine. These results suggest that vaccinia virus may be a more versatile vaccine vector than previously appreciated and that in some instances safety can be prioritized by the complete elimination of viral replication without a proportional loss of immunogenicity.

## Abstract (precis)

The design of viral vectored vaccines is often considered to require a trade-off between efficacy and safety. This is especially the case for vaccines that aim to induce killer (CD8^+^) T cells, where there is a well-established dogma that links infection in vaccinated individuals with effective induction of immunity. However, we found that some proteins of vaccinia virus generate strong CD8^+^ T cell responses even when the virus preparation was inactivated by heat prior to administration as a vaccine. We took advantage of this finding by engineering a new vaccine vector virus that could be used as an inactivated vaccine. These results suggest that vaccinia virus may be a more versatile vaccine vector than previously appreciated and that in some instances safety can be prioritized by the complete elimination of viral replication without a proportional loss of immunogenicity.

## INTRODUCTION

The induction of cytotoxic CD8^+^ T cell responses is important to providing protection against intracellular pathogens, in particular viruses, and for the control of cancer (1, 2). For this reason, it is important to understand the requirements for effective antigen presentation to CD8^+^ T cells to support vaccine design. These requirements can vary across platforms, but some generalizations are thought to hold at least within vaccine types. As an example, for viral vectored vaccines *de novo* expression of the vaccine antigen after administration is considered to be important for generation of CD8^+^ T cell immunity. The reasons for this are that viral gene expression within a dendritic cell (DC) or other antigen-presenting cell (APC) is perhaps the most effective way to deliver antigen for presentation on major histocompatibility complex class I (MHC-I), which is the first requirement for CD8^+^ T cell priming (3). Alternatively, even if a virus does not infect DCs, ongoing infection of other cells delivers a constant supply of viral protein for uptake and cross presentation. In contrast, the generation of robust CD8^+^ T cell responses by replication-incompetent and especially inactivated viral vaccines is more difficult to achieve (4). In general, strong primary and memory CD8^+^ T cell responses do not occur to inactivated vaccines (4–6). Where responses are found, they tend to be orders of magnitude lower than for live virus (7–9). This relationship also fits well with the general paradigm that increasing attenuation results in decreasing immunogenicity (10).

Vaccinia virus (VACV) is well known as the vaccine used to eradicate smallpox, the success of which was underpinned by the high conservation of much of the proteome across these orthopoxviruses (11, 12). In addition, there is increased interest in using VACV as a recombinant vaccine vector to immunize against other viruses and as immunotherapeutics. VACV is an excellent vector for vaccines because it can accommodate up to 25 kb of foreign genome (13), has good stability (14), is well characterized enabling rational attenuation (15), and induces strong responses by all arms of the adaptive immune response (11, 16). VACV has a very dense protein core that encases the dsDNA genome. This protein core is made up of a large number of proteins; however, there are some that are of a particularly high abundance, namely, A3, A4, A10, and A17 (17, 18). The virion core is associated with two lateral bodies, which store viral enzymes to be released into the cell upon infection, the most prominent of which is F17, a phosphoprotein that is also a highly abundant VACV structural protein (17, 18). The lateral bodies and core are wrapped in host-derived membranes and membrane-protruding viral proteins form an entry-fusion complex that drives the early events of cellular infection. In principle, this set of very abundant proteins would be ideal antigens to prime the adaptive immune response, and indeed, the antibody and CD4^+^ T cell response tend to recognize these viral proteins efficiently (16). In contrast, the latest class of VACV genes, which includes virion components is relatively poorly recognized in the CD8^+^ T cell response to infection (11, 16, 19). This is despite no substantial deficit of presentation on infected cells, though there is evidence they might be poorly cross presented from factories in infected cells (19, 20). However, the rules that dictate effective priming after a live infection may differ from those from partially or fully inactivated viruses or virus-infected cells (21, 22). Indeed, the reduction of native VACV epitopes when replication and full viral gene expression are inhibited can lead to improved focusing of the CD8^+^ T cell response on recombinant antigens of interest (21). Another example is where inactivated VACV-infected cells were used to immunize mice as a model of cross presentation. In that case, with the exception of the dominant B8_20_ epitope, the skewing of CD8^+^ T cell responses was toward virion antigens, and some responses were relatively robust (22). This seemed unusual given that, in the experiment shown, the cells were infected for 6 h, at which time the entire viral proteome would be available to prime immune responses and the latest class of antigens has yet to peak (23). This incidental observation from that previous study led us to explore infected cells and then inactivated VACV as an immunogen for CD8^+^ T cells.

Here, we show that some virion proteins prime surprisingly strong responses in mice vaccinated with inactivated VACV. We show that this is not solely the property of particular epitopes or proteins by making recombinant VACVs with virion core proteins tagged with foreign epitopes and finding that these too can be highly immunogenic and protective as inactivated vaccines. Finally, we explored the mechanism by which VACV virions can enter the cytoplasm of an APC. We wondered whether the entry fusion complex might survive inactivation, allowing the viral core to access the cytoplasm for entry into the MHC-I presentation pathway of most cell types. However, we found that only professional APCs, which can cross-present antigens, are able to present epitopes from inactivated VACV virions.

## RESULTS

### VACV virion-associated epitopes are immunogenic after heat inactivation.

**FIG 1:**
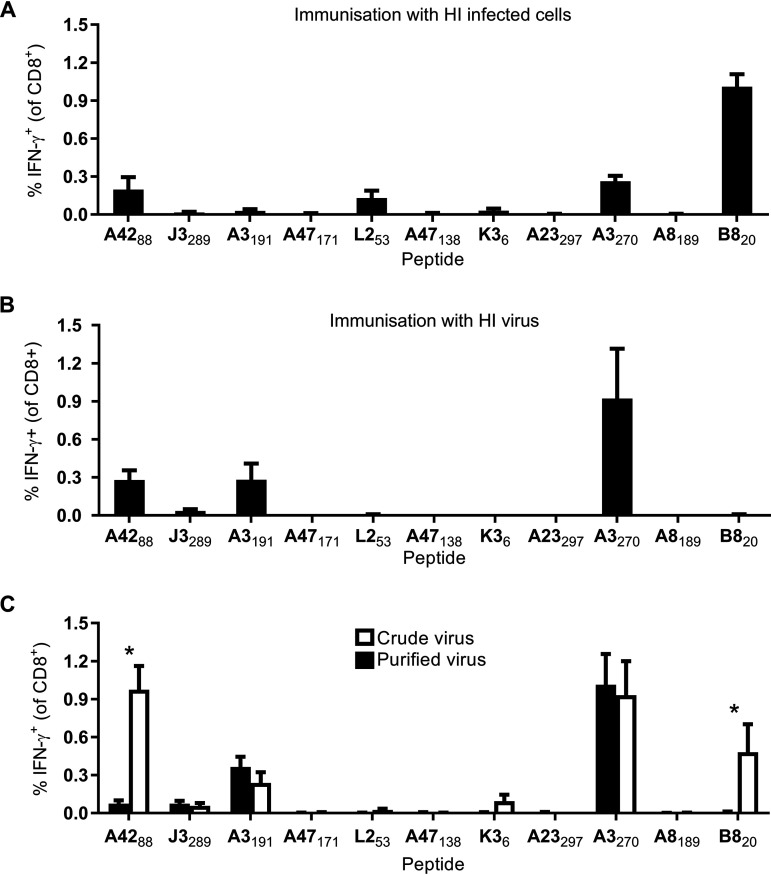
HI VACV virions prime CD8^+^ T cell responses. Mice were immunized by intradermal injection of the ear pinna, and CD8^+^ T cell responses were measured after 7 days. The immunogens were HI VACV WR-infected cells (2 × 10^6^ cells with MOI of 5 PFU/cell for 6 h) (A), HI VACV WR (equivalent of 2 × 10^6^ PFU) (B), and HI crude or highly purified VACV WR (equivalent of 2 × 10^6^ PFU) (C). CD8^+^ T cell responses were measured using a short incubation of splenocytes with the peptides shown, followed by staining for CD8 and intracellular IFN-γ. The means plus the standard errors of the mean (SEM) of data pooled from two independent experiments are shown (*n* = 6). In panel C, an asterisk (*) indicates *P* < 0.05, as determined by ordinary two-way ANOVA and Sidak’s multiple-comparison test. All other values were not significant.

We started this investigation by following up a previous study that found that VACV virion proteins were overrepresented among immunogenic antigens in mice immunized with infected cells. These experiments used recombinant vaccines based on VACV strain WR, but wild-type virus was not tested and infected cells were injected by the intraperitoneal route (22, 24). In our first experiment, MHC-mismatched cells were infected with VACV WR for a total of 6 h, inactivated by heating to 60°C for an hour (here referred to as heat inactivated [HI]), and then used to immunize groups of mice by intradermal injection (25). This published treatment eliminates infectivity and *de novo* viral gene expression and presentation *in vivo*, as shown by the complete loss of responses to VACV-encoded minimal epitope constructs that require direct presentation to prime CD8^+^ T cells (22, 24). The CD8^+^ T cell response to a well-characterized panel of major VACV epitopes was then measured at the peak of the acute response by *ex vivo* stimulation of splenocytes with synthetic peptides and intracellular staining for gamma interferon (IFN-γ) (19, 26–28). A subset of the epitopes that were published as being immunogenic after immunization of HI virus-infected cells were also found to elicit responses here, the difference mostly likely reflecting different routes of immunization (Fig. 1A). B8_20_ was dominant, the next biggest responses were to A3_270_ and A42_88_ (both of with are found in virions), and the remaining response was to the nonvirion epitope L2_53_ (17, 18, 22, 29). Thus, across a set of 11 epitopes that are all immunogenic during a VACV infection (19) and with source antigens that would be present in the infected cells used (23), only two of seven nonvirion epitopes tested were immunogenic, whereas two of four epitopes from virion proteins primed a CD8^+^ T cell response (17, 18, 29).

The above result and past experience in the laboratory that indicated that many VACV virions remain associated with infected cells (D. C. Tscharke, unpublished data) led us to speculate that viral particles themselves may be an efficient source of antigen for priming CD8^+^ T cells. To test this, we used the same heat treatment to make a stock of HI virus and used the equivalent of our usual infection dose to immunize mice. We tested CD8^+^ T cell responses to the same set of 11 VACV epitopes as in the previous experiment, including four derived from virion proteins and seven from nonvirion proteins that require expression *in vivo* to be immunogenic. The seven nonvirion epitopes are critical as controls for virus inactivation (30). Included in this set of negative-control epitopes are B8_20_, K3_6_, A47_171_, and A47_138_, all of which are from early genes, are highly immunogenic, and have been shown to induce CD8^+^ T cell responses to doses as low as 2,000 PFU and when VACV is treated with UV/psoralen to eliminate infectivity, but leaving viral early gene expression intact (19, 22). When the HI virus was used to immunize mice, consistent CD8^+^ T cell responses were generated to the virion-derived epitopes A42_88_, A3_191_, and A3_270_ at levels that were similar to those induced by HI virus-infected cells (Fig. 1B). Importantly, no response was detected to any epitope not present in a virion protein, including B8_20_, K3_6_, A47_171_, and A47_138_, clearly demonstrating effective inactivation of the virus.

Our standard virus preparations as used above are semipurified by centrifugation of cell nucleus-free lysates of infected cells through a sucrose cushion to obtain a pellet of virus. To explore the extent to which cellular debris might contaminate virus preparations and contribute to immunogenicity, the experiment was repeated with virus from a crude cell lysate, as well as a stock of virus that was purified by concentration to a band on a sucrose gradient (Fig. 1C). Some epitopes were only immunogenic (B8_20_ and K3_6_) or more immunogenic (A42_88_) in mice infected with a HI crude virus stock, suggesting that cell debris can be a source of antigen in unpurified preparations. However, the two epitopes from the A3 protein (A3_191_ and A3_270_) were as immunogenic in the highly purified virus as in the crude stock. The size of the response to these epitopes was up to 1% of CD8^+^ T cell in the case of A3_270_, which is not dissimilar to published responses in mice infected with live VACV (19, 27). Notably, A3 is the fourth most abundant virion core protein, estimated to make up >5% of the weight of the virion (17). Finally, neither crude nor purified stocks of virus primed responses to the two epitopes from A47, suggesting that both preparations were adequately inactivated. Taken together, these data suggest that the A3 core protein in VACV virions is a source of antigen that can prime a robust CD8^+^ T cell response even when the virus preparation has been inactivated.

### Epitopes tagged to virion-associated proteins are immunogenic in heat-inactivated VACV.

**FIG 2:**
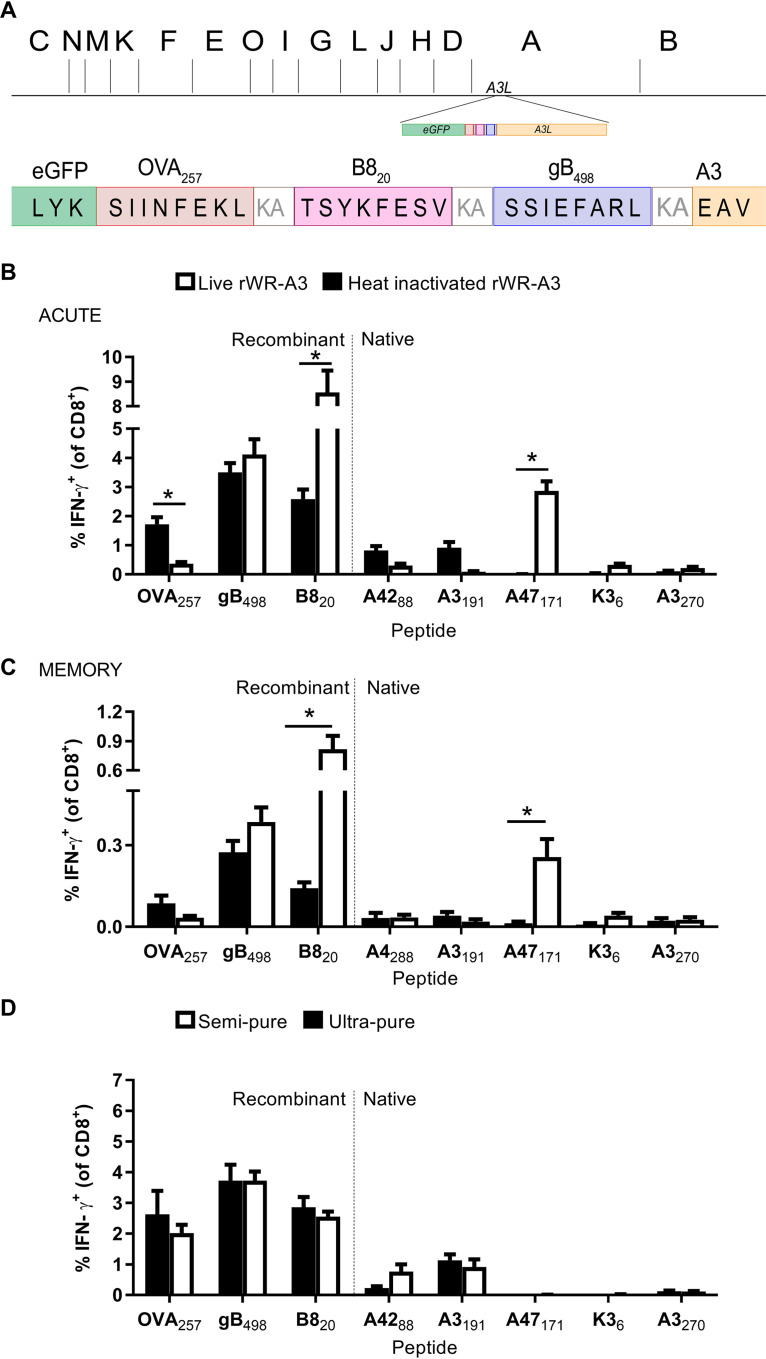
An HI VACV vaccine with epitopes tagged to core antigen A3 elicits strong CD8^+^ T cell responses. (A) Diagram of antigen construct tagged to the N terminus of A3 indicating the position in the genome (shown as HindIII map). (B and C) CD8^+^ T cell responses to live or HI rWR-A3 at 7 days (B) or 7 to 8 weeks (C) after immunization. (D) CD8^+^ T cell responses to standard (semipure) or sucrose gradient (ultrapure) HI rWR-A3 at 7 days postimmunization. The results shown indicate the means plus the SEM of pooled data from at least two independent experiments (B, *n* = 12; C, *n* = 9; D, *n* = 6). *, *P* < 0.05 (determined by ordinary two-way ANOVA and Sidak’s multiple-comparison test; all other values are not significant).

Next, we wanted to know whether the immunogenicity of A3 even after inactivation was unique to the particular epitopes in A3 or could be conferred onto other epitopes fused to this protein. To do this, we created an antigen construct that included enhanced green fluorescent protein (eGFP), and CD8^+^ T cell epitopes from ovalbumin (OVA_257_), VACV B8 protein (B8_20_), and herpes simplex virus (HSV) gB protein (gB_498_). Sequences were added upstream and in frame with the *A3L* gene, and the epitopes were separated by a lysine and an alanine to promote proteasomal degradation at those spacer sites (Fig. 2A), this virus is referred to as rWR-A3.

We first compared the response to the tagged epitopes at an acute time, 7 days after immunization with live or HI rWR-A3. Responses to epitopes from nonvirion proteins were only detectable after live virus immunization, most notably A47_171_, demonstrating that the HI stock was indeed inactivated. However, strong CD8^+^ T cell responses to epitopes within the antigen construct fused to A3 were found in mice immunized with live or HI virus (Fig. 2B). Indeed, for OVA_257_, the response was significantly stronger in mice given the HI virus. The responses to B8_20_ are more complicated to interpret because this epitope is present in two copies: the native context as well as fused to A3. However, with reference to that previous experiment, addition of B8_20_ to A3 has made this epitope immunogenic in the context of HI VACV, whereas the native copy is not in virions and fails to elicit a response when the virus has been killed (compare Fig. 1B to Fig. 2A). Next, we sought to determine whether these apparently strong responses would persist in memory (28 days after immunization). As in the acute response, OVA_257_ and gB_498_ were equally or more immunogenic in mice immunized with HI virus compared to live virus. B8_20_ responses were enhanced in mice given live virus but still present from the HI rWR-A3. The experiments shown above were done using our usual sucrose cushion, semipurified stocks of virus; thus, to confirm that the responses were not due to antigen from any remaining cellular debris, we further purified a portion of these stocks using a sucrose gradient method. CD8^+^ T cell responses to the epitopes tested were the same for both purities, with the exception that the response to A42_88_ was reduced to close to background by the extra purification step.

### CD8^+^ T cell responses generated with heat-inactivated virus are polyfunctional.

**FIG 3:**
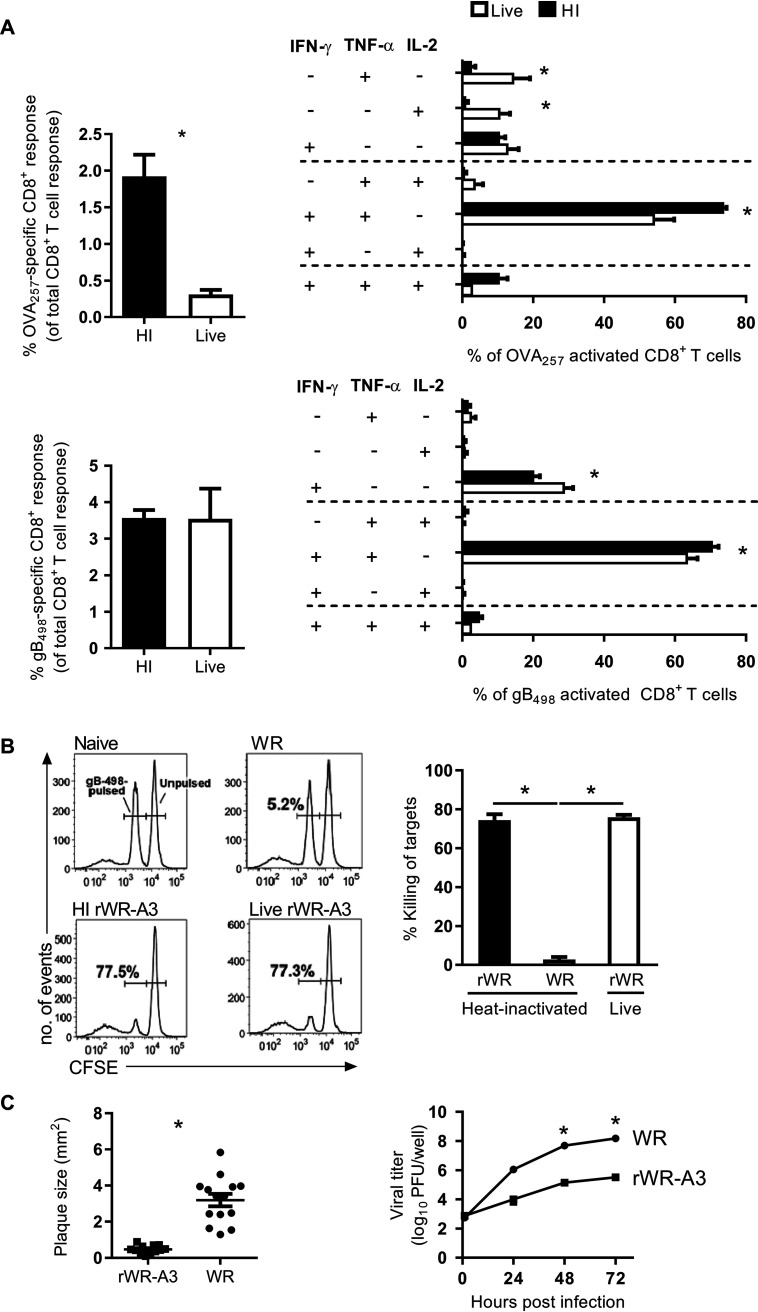
A HI VACV vaccine elicits polyfunctional and cytolytic CD8^+^ T cell responses. (A) Cytokine expression profile of OVA_257_- and gB_498_-specific CD8^+^ T cell responses 7 days after immunization with live or HI rWR-A3, as determined by Boolean analysis using FlowJo and SPICE software. The total responses (left) were calculated as the percentage of cells that express IFN-γ of CD8^+^ T cells. The data are mean plus the SEM of six mice from two independent experiments (*, *P* < 0.05 [Mann-Whitney test, left] or two-way ANOVA and Sidak’s multiple-comparison test [right]). (B) Cytolytic activity of CD8^+^ T cells in mice immunized with live or HI rWR-A3 by *in vivo* cytotoxicity assay against gB_498_-labeled targets. (Left) Representative histograms. Data from six mice across two independent experiments show the means plus the SEM. *, *P* < 0.05 (one-way ANOVA and Dunn’s multiple-comparison test). (C) Plaque size estimation and multiple-step growth curve for rWR-A3 and WR. *, *P* < 0.05 (two-tailed unpaired *t* test [plaque size] or two-way ANOVA and Sidak’s multiple-comparison test for virus growth assay).

Having shown that HI-rWR is capable of eliciting CD8^+^ T cell responses to at least some epitopes at a similar magnitude to that of the live virus, we next wanted to know whether these CD8^+^ T cells would have an equivalent functional capacity. The polyfunctionality of CD8^+^ T cells from mice immunized 7 days earlier was tested by restimulating splenocytes with peptides and examining IFN-γ, tumor necrosis factor alpha (TNF-α), and interleukin-2 (IL-2) expression. Expression of these markers has been shown previously to be characteristic of the immune response to vaccinia virus-based vaccines in humans (31). In accordance with our previous results, the magnitude of the OVA_257_ and gB_498_ responses to HI and live rWR-A3 were greater and similar to live virus, respectively. The proportion of these cells that expressed IFN-γ and TNF-α was greater, when generated with the HI rWR-A3, for both specificities (Fig. 3A). Although the proportion of cells that expressed all three cytokines was not significantly different between the groups of mice and cells expressing just one cytokine were more frequent when live virus was used. We also compared the cytotoxic capability of the CD8^+^ T cell response generated by either the HI or live rWR-A3 or by WR using an *in vivo* cytotoxicity assay. Mice immunized with HI and live rWR-A3 had a similar, strong ability to kill gB_498_-bearing targets (Fig. 3B). Taken together, we interpret these data to show that epitopes tagged to virion protein A3 generate strong CD8^+^ T cell responses that are fully functional in mice immunized with HI virus.

VACVs with N-terminal GFP fusions have been published previously, and these tend to have reduced replication and smaller plaques (32), which matched our casual observation when making and growing rWR-A3. For this reason, we formally quantified plaques and replication compared to the parent, WR. This demonstrated that plaques were significantly smaller and growth significantly reduced for rWR-A3 compared to WR (Fig. 3C).

### Immunogenicity of epitopes when heat inactivated is a property of VACV core proteins in general.

**FIG 4:**
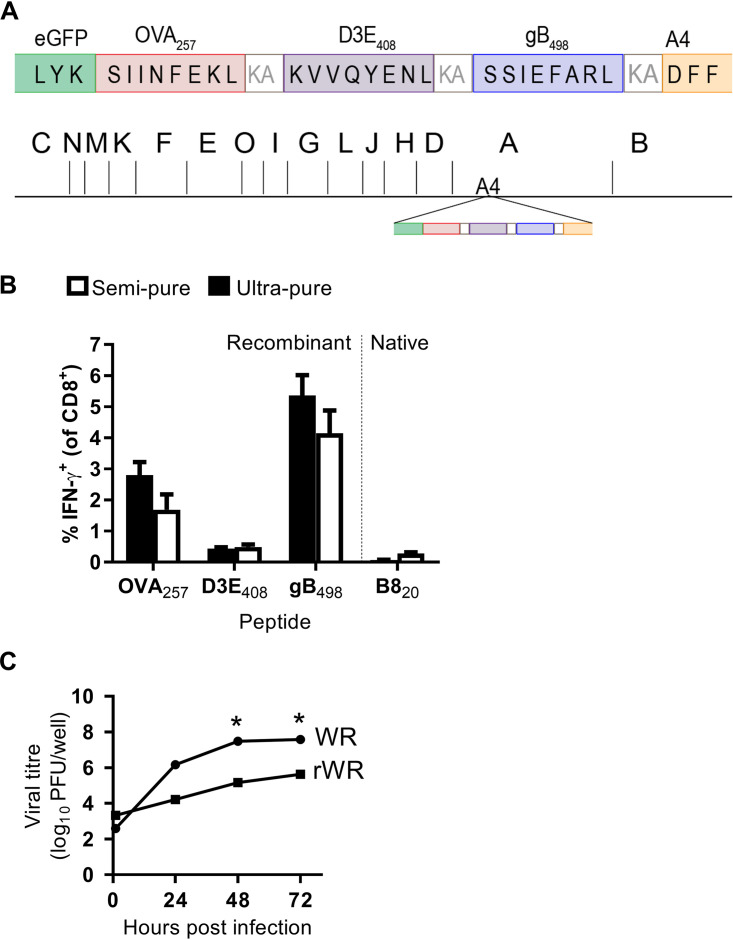
VACV core antigen A4 can be tagged to make a HI VACV vaccine. (A) Diagram of antigen construct tagged to the N terminus of A4 indicating position in the genome (shown as HindIII map). (B) CD8^+^ T cell responses to standard (semipure) or ultrapure (sucrose gradient) HI rWR-A4 at 7 days postimmunization. Means plus the SEM of pooled data from six mice across two independent experiments are shown. (C) Multiple-step growth curve of rWR-A4 and WR. *, *P* < 0.05 (two-way ANOVA and Sidak’s multiple-comparison test) from triplicate cultures.

Having showed that native and recombinant epitopes processed from A3 generate good CD8^+^ T cell responses even when inactivated, we wanted to determine whether this might also be the case for other core proteins or was perhaps related to the particular structural characteristics of A3. This was especially the case given that our A3 virus grew poorly and that this protein has been identified as immunoprevalent, a frequent source of epitopes, and immunogenic across many MHC allomorphs in multiple species (33, 34). We chose A4 because like A3 it is highly abundant in the virion core, being estimated to comprise around 24% of the weight of virions (17, 35) but, unlike A3, while CD8^+^ T cell epitopes have been predicted in A4 (36), none have been identified in the context of VACV infection in humans or mice. The virus we made was called rWR-A4 and had an eGFP-antigen construct tagged to the N terminus of *A4L*, which was similar to the one used above for A3 but has a dengue virus D3E epitope (D3E_408_) between OVA_257_ and gB_498_ instead of B8_20_ (Fig. 4A). Just as for rWR-A3, this virus could prime strong CD8^+^ T cell responses to the recombinant epitopes, even when inactivated by heat, and this result was seen for semipurified and purified virus stocks (Fig. 4B). In this experiment, responses to the nonvirion epitope B8_20_ were not detected, demonstrating effective inactivation of the HI virus stock. Also similar to rWR-A3L, the growth of rWR-A4 *in vitro* was substantially reduced compared to WR (Fig. 4C). These results show that multiple VACV virion proteins can be immunogenic for CD8^+^ T cells when delivered as an HI vaccine.

### Epitopes can be tagged to lateral bodies without compromising replication.

**FIG 5:**
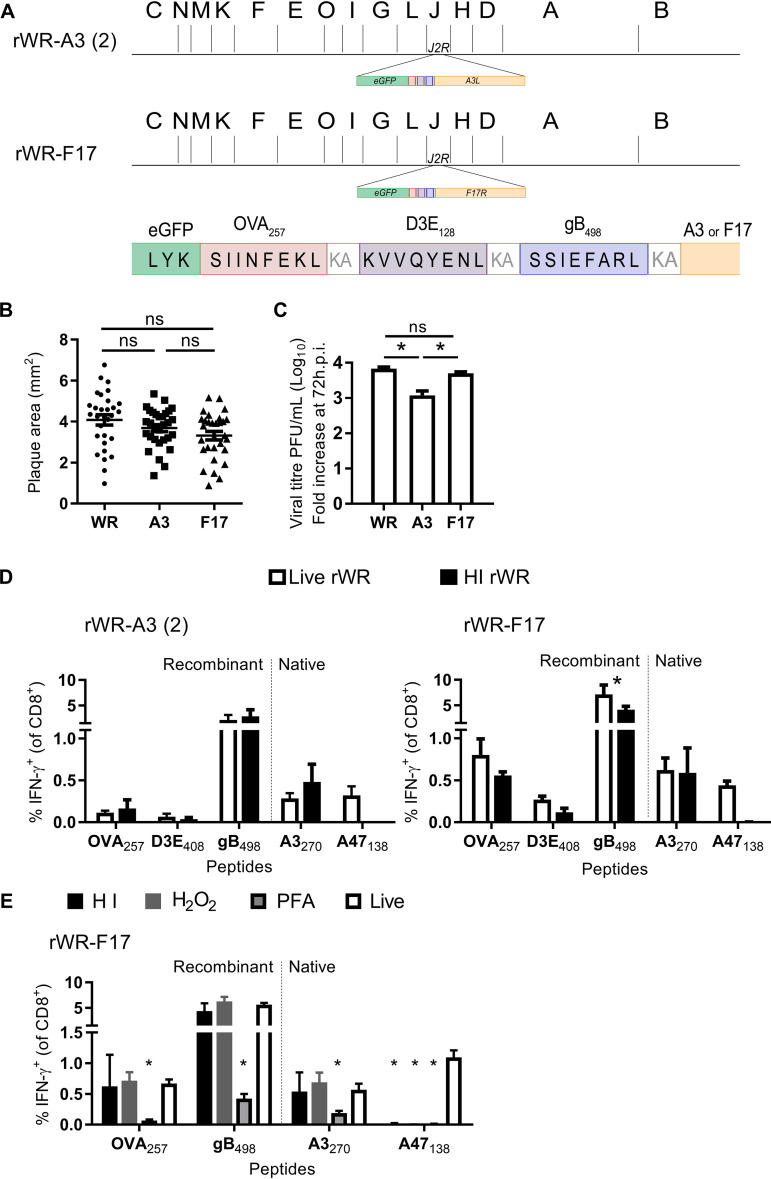
VACV with epitopes tagged to lateral body protein F17 is attenuated in culture and elicits strong CD8^+^ T cell responses as an HI vaccine. (A) Diagram of antigen construct tagged to the N terminus of *A3L* or *F17R* indicating the position of the insertion in the genome (shown as HindIII map). (B and C) Plaque size (B) and growth (C) in cultures of rWR-A3(2), rWR-F17, and nonrecombinant WR. *, *P* < 0.05 (one-way ANOVA and Dunn’s multiple-comparison test; others ns). (D) CD8^+^ T cell responses 7 days after immunization of mice with live and HI rWR-A3(2) and rWR-F17. The data are mean+SEM of 9 mice from 3 experiments; *P < 0.05 two-way ANOVA and Tukey’s multiple-comparison test. (E) CD8^+^ T cell responses 7 days after immunization of mice with live rWR-F17 or the same virus inactivated with heat (HI), H_2_O_2_ or paraformaldehyde (PFA). The data are from 10–12 mice. *, *P* < 0.05 (calculated as for panel C against values from mice immunized with live virus; other values are not significant).

Having shown that tagging VACV core proteins using the native copy of the gene results in good CD8^+^ T cell responses from heat-killed virus but compromises replication, we wanted to find a potential vaccine of this type that grew with wild-type kinetics. To do this, we tried a strategy of tagging our antigen construct to an extra copy of the core gene inserted in the thymidine kinase (TK) gene (*J2R*). The rationale was that virions would then have a mix of tagged and unmodified protein, which might mitigate the impact of the tag on protein function. In addition to a new version of our A3-tagged virus [rWR-A3(2)], we used this approach to extend our findings to the tagging of the lateral bodies by modifying F17 to make rWR-F17. F17 is also highly abundant, accounting for ∼7.5% of virion weight, but it does not have a clear structural role like A3 and A4, so we reasoned that tagging this protein might have less impact on virion morphology and therefore virus replication (17). The new rWRs carried eGFP, followed by OVA_257_, D3E_408_, and gB_498_, as an antigen tag to the extra copy of *A3L* or *F17R* in the *J2R* gene region (Fig. 5A).

First, we tested plaque size and replication and found no statistically significant reduction in plaque size for either rWR-A3(2) or rWR-F17 compared to WR (Fig. 5B). In terms of replication, WR-A3(2) had a significant defect, but the growth of rWR-F17 was the same as WR over 72 h in a multiple-step growth experiment (Fig. 5C). These viruses were then used to immunize mice as live and HI vaccines. Consistent with previous results, HI rWR-F17 and HI rWR-A3(2) were able to prime CD8^+^ T cell responses to the tagged epitopes at levels that were similar to the corresponding live virus, with the exception of gB_498_ from rWR-F17 (Fig. 5D). Finally, we were interested in whether the preservation of immunogenicity was a particular characteristic of heat inactivation. For this reason, we treated rWR-F17 with heat, H_2_O_2_, or paraformaldehyde to inactivate the virus before immunizing mice and then measuring CD8^+^ T cell responses 7 days later. HI- and H_2_O_2_-treated virus was effective as a vaccine, eliciting responses to virion epitopes that were similar to those induced by live rWR-F17, but paraformaldehyde-treated virus was a significantly poorer vaccine, eliciting responses that were roughly 10-fold lower than the other formulations (Fig. 5E). All of the experiments described here used A47_138_ as a nonvirion epitope to control for the effectiveness of virus inactivation and, in all cases, strong CD8^+^ T cell responses were found to this epitope when virus was live but not inactivated.

### HI prime and live boost results in optimal reduction in HSV pathogenesis.

**FIG 6:**
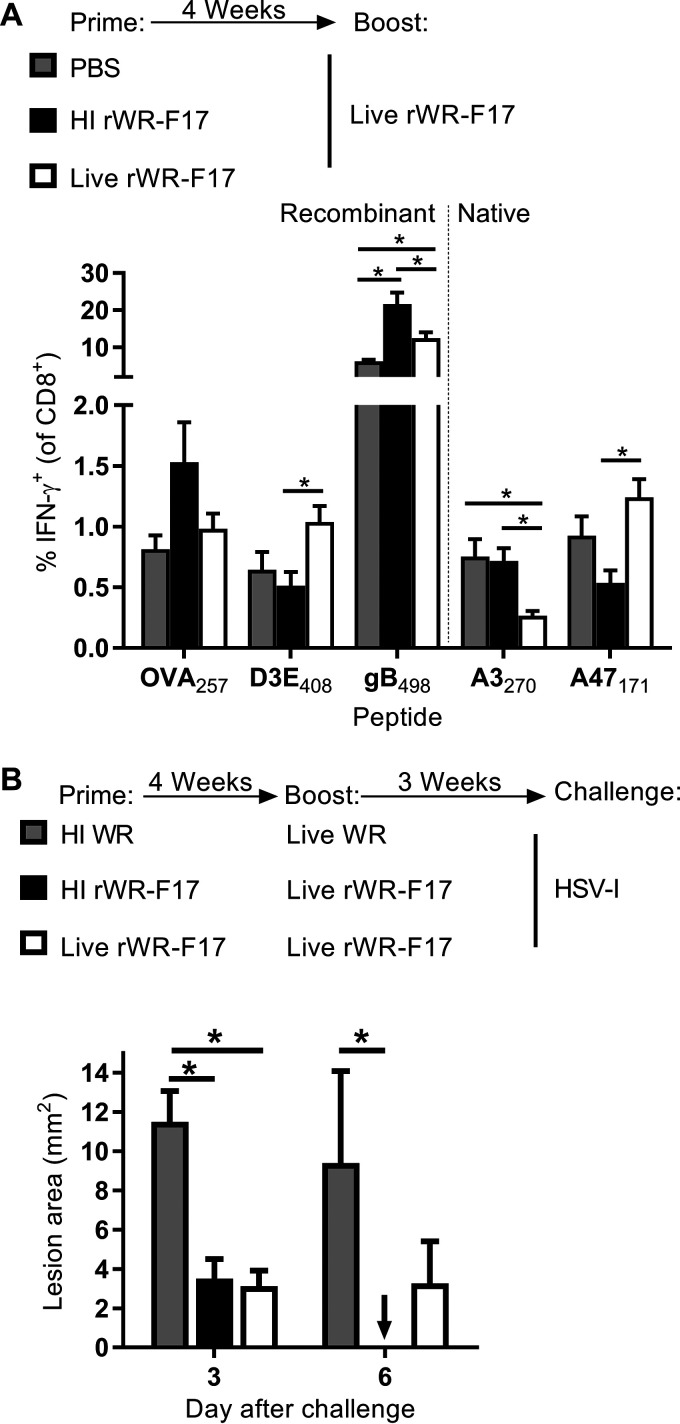
Prime-boost immunization with HI and live rWR-F17 is immunogenic and is effective against HSV. (A) Mice were primed with the vaccines shown and then boosted with live rWR-F17 after 4 weeks, and CD8^+^ T cell responses were measured 7 days later. The data are means plus the SEM of nine (PBS prime) or ten (all others) mice pooled from two experiments. (B) Mice were primed and boosted as shown in panel B but challenged after 3 weeks with HSV-1 by tattooing, and the lesion area was measured on the days indicated. The data are means plus the SEM from eight mice and were pooled from two experiments. *, *P* < 0.05 (two-way ANOVA with Tukey’s multiple-comparison test; other values are not significant).

VACV vaccines are often given in prime-boost regimes, so we wondered whether HI virus might make a good priming agent to be boosted by the same virus given as a typical live vaccine. Mice were primed with live or HI rWR-F17 or given phosphate-buffered saline (PBS) then boosted with live rWR-F17 after 4 weeks and CD8^+^ T cell responses were measured 7 days later. For OVA_257_ and gB_498_, the mean magnitude of response was higher for the mice primed with HI virus compared to the other two groups, and this was statistically significant for gB_498_ (Fig. 6A). D3E_408_ behaved differently with the live boost being superior; however, we note that this epitope was always only weakly immunogenic from HI viruses. Interestingly for the two native VACV epitopes, the live prime favored responses to the early gene A47, apparently at the expense of A3, but priming with HI virus gave the opposite response. These data are reminiscent of the favoring of early expressed antigens in a secondary infection with VACV and the boost to responses for an epitope primed previously by itself that have been noted in the literature (37, 38). The repression of A47 responses by priming with HI virus also shows that the stock used for this experiment was adequately inactivated.

Having found rWR-F17 to be immunogenic in prime-boost strategies, we wanted to test whether it might also be protective. After prime boosting with different combinations of live or HI rWR-F17 or WR (as a control), mice were infected with HSV by tattoo, and lesion development was measured. On day 3, both groups of mice that were boosted with rWR-F17 had smaller lesions than the control group that had an irrelevant (WR) boost, but only mice that received the HI rWR-F17 prime dose resolved their lesions by day 6, a result that differed significantly from the control (Fig. 6B). Together, these data demonstrate that a strategy of using HI rWR-F17 and boosting with the same virus given live a boost can be immunogenic and effective, at least in the context of gB_498_ and HSV.

### HI VACV virions are efficient substrates for regular cross presentation.

The surprising immunogenicity of HI VACV virion antigens suggests that the presentation of epitopes from these virions must be particularly efficient, perhaps due to use of a unique mechanism. For antigens to be presented on the APC cell surface on MHC-I, they must be present in the cytosol for processing and transport into the endoplasmic reticulum. The deposition of HI virions into the cytoplasm could be occurring by one of two mechanisms: either by regular cross presentation or potentially by virus-mediated entry if the VACV entry and fusion proteins remain intact. Our speculation was that if HI virions were able to enter APCs via a viral entry process, this might explain the excellent immunogenicity given that viral infection is typically a very efficient process.

**FIG 7:**
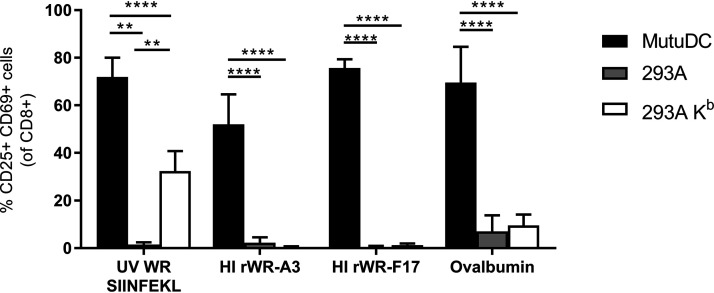
Presentation of epitopes from HI virus requires DCs. Cell lines were cultured with antigens and viruses as shown in the legend and *x* axis, respectively, for 24 h and cocultured with OT-I CD8^+^ T cells for a further 24 h. Activation of OT-I CD8^+^ T cells was determined by measuring upregulation of CD25 and CD69 by flow cytometry. The data are means plus the SEM from at least four pooled independent experiments. ****, *P* < 0.0001; **, *P* < 0.01 (two-way ANOVA with Tukey’s multiple-comparison test).

We reasoned that if HI virions retained their entry capacity, any cell usually permissive for VACV infection should be able to present virion epitopes on MHC-I. Conversely, if cross presentation was required, presentation of virion epitopes would be restricted to DCs. Therefore, to determine the mechanism by which HI VACV virions can be presented, we compared activation of OT-I CD8^+^ T cells after coculture with three different cell types exposed to HI VACV and controls: (i) MutuDCs, a DC cell line capable of cross presentation; (ii) 293KbC2, a fibroblast line expressing H-2K^b^ that is permissive for VACV infection and can present OVA_257_; and (iii) 293A cells, which can be infected but cannot present OVA_257_ as a negative control. The controls were a WR that expresses OVA_257_ as a minimal antigen construct and given a brief UV treatment (to ablate replication, but not antigen expression) (39) and ovalbumin protein. The control treatments behaved as expected, with ovalbumin only activating the OT-1 cells when given to MutuDCs, but the UV-irradiated WR expressing the minigene was able to present OVA_257_ from the DCs and 293K^b^ cells (Fig. 7). The HI VACVs behaved like ovalbumin protein, only being able to prime the OT-Is after incubation with MutuDCs, demonstrating that the internalization of the HI rVACV and presentation of virion epitopes is a DC-specific process. Thus, the best explanation for the robust priming of CD8^+^ T cells we saw *in vivo* is that VACV virions are very efficient substrates for regular cross presentation onto MHC-I by DCs.

## DISCUSSION

Here, we show that, in contrast to dogma for viruses and viral vectors, *de novo* antigen expression is not necessarily required for the generation of strong CD8^+^ T cell responses to VACV as long as the epitopes are included in the virion core. In addition, we took advantage of this finding and engineered novel poxvirus vaccine vectors with CD8^+^ T cell epitopes tagged to core epitopes. These vaccines could prime polyfunctional responses that are retained into memory and when combined with a live boost were effective against a viral challenge.

Efficient CD8^+^ T cell priming without viral gene expression in the host occurred with nonrecombinant WR and all of our recombinant viruses. T cell priming occurs when the interaction, or sequential interactions between a DC presenting the antigen on MHC-I and the CD8^+^ T cell with cognate T cell receptor (TCR). There are multiple steps in the antigen presentation and T cell priming processes that would be expected to be limiting in the absence of viral gene expression. The most obvious of these is the small amount of viral protein present when viral replication is precluded; only the initial inoculum is available. Next, there cannot be engagement of the classical MHC-I presentation pathway, which is fed largely by the products of recent translation (40–45). Finally, there will be a reduction in tissue damage and therefore damage associated molecular patterns that may boost DC maturation. All of these would predict fewer activated APCs, each presenting fewer viral epitopes. Our findings suggest that all of these limitations are overcome in the case of epitopes that are present in VACV virions. We speculate that inherent structural properties of the VACV virions facilitate effective uptake by DCs and efficiently deliver a large amount of antigen to the cross-presentation pathway. In this context, the large virion with some components present at a high copy number is likely to be important, and we considered this when choosing core proteins to tag when engineering our recombinant viruses. For example, multiple groups using a variety of methods found that F17, A3, and A4 are of the most abundant proteins found in the mature virion (17, 18, 46). Indeed, F17, A3, and A4, together with A10 and A17, make up 80% of the proteins within the WR core (18), highlighting the density of these proteins in the virion. Coupled with a relatively large virion size, this density presumably allows delivery of many copies of these proteins to DCs. Having noted these specifics about VACV, virion proteins from other viruses can be presented, so the phenomenon may be more widespread than appreciated, and virion proteins may be an overlooked source of CD8^+^ T cell epitopes (47, 48).

Priming of CD8^+^ T cells by virion antigens from HI virus may have some advantages. VACV is a large virus with many antigens that may compete for presentation and CD8^+^ T cell priming. In contrast, these are limited to just those present within virions for HI VACV, reducing the number of potential viral antigens by two-thirds from >200 to ∼80. Taking into account that many of these proteins are only minor constituents and that we can target epitopes to the abundant structural proteins, the advantage of reduced competition can be made even greater (19). This would reduce CD8^+^ T cell competition for access to DCs, potentially favoring responses to virion, and in our case vaccine-specific epitopes. This can be especially important in prime-boost strategies where initial immunization with HI rVACV can generate large pools of CD8^+^ T cells toward the vaccine epitopes that then have an advantage in a subsequent live VACV boost. We note that, for HI VACV, we eliminate presentation of the immunodominant B8_20_ epitope and other strategies that reduce responses to this peptide tend to allow greater expansion of CD8^+^ T cells primed by less dominant specificities (27, 28). In our prime-boost experiment (Fig. 6A), responses to A47_171_ were reduced in mice primed with HI rWR-F17, suggesting that prior priming of virion epitopes with an inactivated virus reduces responses to nonvirion antigens. Whereas previously we noted priming with single peptides did not alter responses to other native VACV epitopes (38). Regardless, this is a practical example of how we are able to engineer a viral vector with the aim of limiting responses toward vector antigens and promoting responses toward the vaccine epitopes. This has been achieved by others using UV inactivation to treat a virus expressing a minimal antigen construct, which eliminates most virus gene expression and replication but spares the epitope of interest (21). The advantage of using HI VACV over the UV strategy is that we can add larger antigens. Finally, we note that we provide here the first example of a prime-boost immunization strategy wherein only one recombinant virus (or biological) needs to be made, but the benefits of having different vectors are retained.

The responses generated by our HI rWRs were so robust that they were still present at memory time points and were activated in a recall infection and expressed multiple cytokines. The generation of memory CD8^+^ T cell responses highlights the efficient nature of priming with our HI rWR. Beyond improving the position of a vaccine antigen of choice in an immunodominance hierarchy, there may be some advantage to the reduced levels of antigen and less-inflammatory environment provided by HI VACV, because that quality of response and extent of memory can be favored where antigen and inflammation are limited (49–53). Further, the many VACV immunomodulatory genes will not be effective when the virus is inactivated. Finally, we note that we have not explored the use of any adjuvants and that these might further improve the immunogenicity of HI VACV. Likewise, the use of HI VACV in prime-boost strategies with other agents could be investigated.

We explored the mechanism of presentation of the virion epitopes to see whether HI virions might retain the ability to enter cells through the usual infection process, although this seemed unlikely, we wondered whether such effective priming of CD8^+^ T cells might be underpinned by a unique mechanism. Further, VACV late antigens as present in factories in infected cells have been found to be poor substrates for cross presentation on MHC-I and virion uptake and presentation of antigens on MHC-II by the classical exogenous pathways has been shown to be very inefficient (20, 54, 55). In contrast, we found that DCs (and only DCs) were able to present epitopes from HI virions on MHC-I, and this suggests that a DC-specific process, most likely regular cross presentation, is required. Indeed, the efficient routing of virions to an MHC-I cross-presentation pathway may explain why they have such poor access to the canonical pathway taken by most exogenous antigens for presentation on MHC-II (54). The result of such a switching of presentation pathway would be efficient priming of CD8^+^, rather than CD4^+^ T cells, and not vice versa as would be conventionally expected (54). These findings suggest that it is worth probing the cell biology of antigen processing of virions in general. Virions have unique properties in terms of size, periodicity of subunits, and the way they disassemble, which means they probably represent a separate class of antigens distinct from others, such as cell-associated, aggregated, or soluble forms.

Historically, vaccinia virus is well known as the immunization agent in the eradication of smallpox. Genetic engineering has not only improved the use of VACVs in immunizing against orthopoxviruses but has allowed them to be used as vectors for the delivery of CD8^+^ T cell epitopes from other viruses. Here, we were able to create recombinant VACVs that utilized several viral core proteins as the carriers of foreign epitopes. In doing so, we were able to inactivate the viruses but still deliver the CD8^+^ T cell epitope in the virus particle and thus induce CD8^+^ T cell responses to these antigens. For most experiments, we used heat, but we also found that H_2_O_2_ is effective, and this has been suggested more broadly as a useful inactivation agent that maintains the integrity of antibody epitopes (8). With this in mind, it may be possible to create a killed recombinant VACV vaccine that activates strong humoral and CD8^+^ T cell responses if the surface of the virion were able to be decorated with an antigen of choice. Such a vaccine would capitalize on the inherent properties of VACV virions, including the activation of DCs, and the capacity to be efficiently cross presented at the same time as having enhanced safety due to inactivation.

## MATERIALS AND METHODS

### Mice.

Female, specific-pathogen-free C57BL/6 mice were used at between 7 and 14 weeks of age for all experiments. The mice were obtained from the Australian Phenomics Facility (Canberra) or the Animal Resources Centre (Perth). All experiments were conducted in accordance with the ethics protocols (F.BMB.38.08, A2011.001, A2013.037, and A2016.045), which were approved by the Australian National University Animal Ethics and Experimentation Committee.

### Cells and virus.

For assays where cells were infected before being used to immunize mice, 293A cells (ATCC, CRL-1573) were used. For *in vitro* viral growth or plaque assays, BSC-1 cells (ATCC, CCL26) were used. For antigen presentation assays; MutuDCs (originally a gift from Hans Acha-Orbea, Lausanne, Switzerland) (56), 293A cells, and 293KbC2 cells (57) were used. Unmodified WR was originally a gift from B. Moss (National Institutes of Health, Bethesda, MD). HSV-1 strain KOS was provided by F. Carbone (University of Melbourne, Melbourne, Australia). Both were grown and titrated according to standard methods. Where indicated, virus or virus-infected cells were diluted in PBS and heat inactivated by incubation at 60°C for 1 h. Alternative inactivation methods included treatment of concentrated stocks with H_2_O_2_ as published (8) and with 1% paraformaldehyde for 20 min.

### Generation of recombinant viruses.

All of the viruses used are listed in Table 1, along with descriptions of any modifications. All additional antigens were expressed with the promoter native to the VACV open reading frame described. The viruses were made with homologous recombination between transfected transfer plasmids and the WR genome as previously described (58). Transfer plasmids were based on pSSmCB, and details of the corresponding recombinant viruses are presented in Table 1. Briefly, 293A cells were infected with WR at a multiplicity of infection (MOI) of 0.05 in Dulbecco modified Eagle medium (DMEM) supplemented with 2% fetal bovine serum (FBS). After 1 h, the inoculum was replaced with a transfection mixture consisting of 1% Lipofectamine 2000 (Life Technologies), the plasmid of interest, and DMEM. After 2 days, the virus was released by multiple freeze-thaw cycles and sonication. Recombinant viruses were isolated by serial steps of plaque purification on BSC-1 cells and transient dominant selection using mCherry/blasticidin resistance gene expression. Isolated plaques were analyzed by diagnostic PCR and sequencing for the recombinant regions to confirm insertions.

**TABLE 1:** VACVs used

Name^*a*^	Full name^*b*^	Description	Source or reference
WR	WR	Wild-type VACV strain Western Reserve (WR)	63
WR mini OVA	WR-TK-ESminiOVA	WR with SIINFEKL gene inserted within the TK region	64
rWR-A3	WR-eGFP-STS-A3	WR with eGFP-STS antigen construct inserted in-frame with the A3 gene	This study
rWR-A4	WR-eGFP-SKS-A4	WR with eGFP-SKS antigen construct inserted in-frame with the A4 gene	This study
rWR-A3(2)	WR-eGFP-SKS-A3	WR with eGFP-SKS antigen construct inserted in-frame with a duplicate A3 gene in the TK region	This study
rWR-F17	WR-eGFP-SKS-F17	WR with eGFP-SKS antigen construct inserted in-frame with a duplicate F17 gene in the TK region	This study

a Viruses are listed in the order they appear in the text.

b The full virus name includes the parental virus (WR), eGFP, and the first letter (amino acid) of each inserted epitope and the VACV protein tagged.

### Virus purification methods.

Unless stated otherwise, the virus stocks were standard sucrose cushion (semipurified) preparations. According to this method, infected cells are resuspended in 10 mM Tris (pH 9), the plasma membranes are ruptured with a Dounce homogenizer, and nuclei are removed by centrifugation. The supernatant containing the virus was layered onto a 36% sucrose cushion in 10 mM Tris (pH 9) and then, after ultracentrifugation, the pellet containing the virus was resuspended in 10 mM Tris (pH 9). Crude virus stocks refer to a preparation made by suspending infected cells in 10 mM Tris (pH 9) before three freeze-thaw cycles and sonication to release the virus, but no further steps were performed to remove cell debris. Highly purified virus stocks were made by layering a semipurified (sucrose cushion) stock of virus onto a sucrose gradient and ultracentrifugation at 12,000 rpm for 50 min. The layer of purified virus midway down the gradient was isolated and resuspended in 10 mM Tris (pH 9). This virus was further concentrated by centrifugation, and the pellet was resuspended in 10 mM Tris (pH 9).

### Virus growth *in vitro* and plaque size estimation.

For measuring *in vitro* growth, confluent monolayers of BSC-1 cells in six-well plates were incubated with either rWR or WR inoculum at an MOI of 0.01 PFU/cell for 1 h at 37°C and 5% CO_2_. Next, the inoculum was replaced with DMEM supplemented with 2% FBS, and the virus cultures were incubated at 37°C and 5% CO_2_ for the times indicated. Virus was harvested by scraping cell monolayers, centrifugation at 2,000 rpm for 10 min, and resuspension. The cell pellet was resuspended in DMEM supplemented with 2% FBS and subjected to three freeze-thaw rounds. Virus was titrated according to standard methods wherein infected monolayers were stained with crystal violet. Plaques were counted to determine titer, and their sizes were estimated using ImageJ.

### Infection and immunization of mice.

Mice were anesthetized with isoflurane (4% in O_2_) inhalation. Anesthetized mice were immunized by intradermal injection with 10 μl of inocula containing 2.0 × 10^6^ VACV PFU (either live or heat inactivated) or 1.0 × 10^7^ infected cells in PBS into the left ear pinnae (25, 59, 60). For prime-boost vaccination regimes, mice were intradermally immunized (as indicated in Fig. 6) with 10 μl of either PBS or 2.0 × 10^6^ PFU of live or HI rWR-F17. At 4 weeks after priming, the boost dose was administered as follows: 10 μl of inoculum by intradermal immunization of 2.0 × 10^6^ PFU of live WR or rWR-F17. For CD8^+^ T cell response assays, mice were sacrificed, and splenocytes were isolated 7 days after the final immunization. For HSV-1 challenge experiments, mice were infected with HSV-1 strain KOS as described below, 7 days after immunization with the boost dose.

### Measurement of CD8^±^ T cell response to epitopes.

CD8^+^ T cell responses were measured in spleens as previously described (26, 61). To restimulate and measure the CD8^+^ T cell response, 1.0 × 10^6^ splenocytes were cultured in 0.1 μM synthetic peptide (GenScript, Piscataway, NJ) or Mimotopes (Clayton, Victoria, Australia; Table 2) for a total of 4 h, with brefeldin A added to a concentration of 50 μg/ml after the first hour. The cells were then labeled for CD8 (anti-mouse CD8α-PE; BioLegend, clone 53.67) diluted in PBS with 2% FBS and intracellular IFN-γ (anti-mouse IFN-γ-APC; BioLegend, clone XMG1.2) diluted in PBS with 2% FBS and 0.25% saponin. Events were gated sequentially on SSC × FSC (lymphocytes), SSC-H × SSC-W (single cells), FSC-H × FSC-W (single cells), SSC × PE (CD8^+^ cells), and PE × APC (IFN-γ^+^ CD8^+^ cells) plots to determine the percentages of CD8^+^ T cells that were IFN-γ^+^. To assess CD8^+^ T cell polyfunctionality, experiments were performed as described above, and cells were also labeled for TNF-α (anti-mouse TNF-α-PE-Cy7; BD Pharmingen, clone MP6-XT22) and IL-2α (anti-mouse IL-2α-PE-Cy5), both diluted in PBS with 2% FBS and 0.25% saponin. Boolean gating was performed using FlowJo software, and data were analyzed using SPICE software (National Institutes of Health).

**TABLE 2:** Synthetic peptides

Peptide	Origin^*a*^	Sequence	MHC	Class^*b*^	Virion^*c*^	Reference(s)
A42_88_	VACV, A42, 88–96	YAPVSPIVI	H-2D^b^	I	Y, 0.84	57
J3_289_	VACV, J3, 289–296	SIFRFLNI	H-2K^b^	E1.2	Y, 0.59	65
A3_191_	VACV, A3, 191–199	YSPSNHHIL	H-2K^b^	I	Y, 5.55	65
A47_171_^*d*^	VACV, A47, 171–180	YAHINALEYI	H-2K^b^	E1.2	N	66
L2_53_	VACV, L2, 53–61	VIYIFTVRL	H-2K^b^	E1.1	N	65
A47_138_	VACV, A47, 138–146	AAFEFINSL	H-2K^b^	E1.2	N	57
K3_6_	VACV, K3, 6–15	YSLPNAGDVI	H-2D^b^	E1.1	N	57
A23_297_	VACV, A23, 297–305	IGMFNLTFI	H-2D^b^	E1.2	N	65
A3_270_	VACV, A3, 270–277	KSYNYMLL	H-2K^b^	I	Y, 5.55	65
A8_189_	VACV, A8, 189–196	ITYRFYLI	H-2K^b^	E1.1	N	65
B8_20_	VACV, B8, 20–27	TSYKFESV	H-2K^b^	E1.1	N	57
OVA_257_	Chicken, ovalbumin, 257–264	SIINFEKL	H-2K^b^			67, 68
gB_498_	HSV-1, glycoprotein B, 498–505	SSIEFARL	H-2K^b^			69
D3E_408_	Dengue virus type 3, envelope protein E, 408–415^*e*^	KVVQYENL	H-2D^b^			70

a Origin listings indicate the organism or virus of origin, the protein from which the epitope is derived, and the residue numbers within that protein.

b Kinetic class for VACV genes: E1.1 and E1.2, early; I, intermediate.

c For VACV proteins only: N, nonstructural; Y, virion component (numbers indicate the estimated percent virion weights from reference 17).

d Synthesized by Mimotopes.

e Residue numbers refer to position within dengue virus type 3 polyprotein, as previously published in (70).

### HSV challenge.

Mice were anesthetized with intraperitoneal injection of Avertin (1,1,1-tribromoethanol in 2-methyl-2-butanol) and infected with HSV-I strain KOS by tattoo on the left flank as previously described (62). After shaving and depilation, a round shader needle was dipped in HSV at a concentration of 1 × 10^8^ PFU/ml for 10 s. A 5-mm × 5-mm square at the tip of the spleen was tattooed for 10 s, and the excess inoculum was wiped off. Mice were monitored, and the lesion area was estimated on days 3 and 6 after challenge.

### *In vivo* CTL assay.

The *in vivo* CTL assay was performed as previously described (28, 61). Briefly, splenocytes were harvested from a naive mouse and labeled with Vybrant DiD cell-labeling dye (Life Technologies). Splenocytes were pulsed with either 0.1 μM gB_498_ peptide for 1 h at 37°C or remained unpulsed in DMEM without FCS. After a washing step, the cells were stained with 0.25 μM (peptide pulsed; CFSE-low) or 2 μM (unpulsed; CFSE-high) CFSE (carboxyfluorescein succinimidyl ester). The pulsed and unpulsed cells were counted, mixed at a 1:1 ratio, and resuspended in PBS. A total of 1.0 × 10^7^ cells in 200 μl was injected into the tail veins of naive mice or mice that had been immunized with HI or live rWR-A3 or with HI WR 7 days previously. Ar 4 h after cell transfer, spleens were collected, and splenocytes were analyzed by flow cytometry. The transferred cells were first identified by Vybrant DiD staining, and then the proportions of cells that were CFSE-high or CFSE-low were determined. The lysis ratio was calculated as follows: ratio = (% CFSE-low/% CFSE-high), for either a naive or an immunized mouse. The percent killing was determined as follows: % killing = [1 – (lysis ratio within a naive mouse)/(lysis ratio within an immunized mouse) × 100], as previously described (28).

### *In vitro* antigen presentation assay.

HI rWR (A3 or F17 where indicated) or UV-attenuated WR-mini-OVA was used to infect either MutuDCs, 293A cells, or 293KbC2 cells (57) for 2 h with rocking at an MOI of 10 before the inoculum was replaced with IMDM-10. Splenocytes from a naive OT-I mouse were harvested and subjected to CD8α-negative enrichment (Miltenyi Biotech, 130-096-543). The eEnrichment efficiency was checked by flow cytometry, and samples were at least 85% CD8α^+^ Vα2^+^ T cells. OT-I CD8^+^ T cells were added to V-bottom plates in D10 with β-mercaptoethanol, and infected cells were added in the same medium at a ratio of 1:2 (infected cell target to OT-I). After 24 h, the cells were labeled with anti-mouse CD11c-FITC (BioLegend, clone N418), anti-mouse CD8α-PE-Cy7 (BioLegend, clone 53.67), anti-mouse TCR Vα2-APC (BioLegend, clone B20.1), anti-mouse CD69-PerCP-Cy5.5 (BioLegend, clone H1.2F3), and anti-mouse CD25 (BioLegend, clone 3C7) antibodies diluted in PBS with 2% FBS. Events were gated sequentially on SSC-A × FSC-A (lymphocytes), FSC-H × FSC-W (single cells), SSC-H x SSC-W (single cells), SSC-A × FITC/GFP^–^ (MutuDC exclusion), PE-Cy7 × APC (CD8^+^ Vα2^+^ cells), and PE × PerCP-Cy5.5 (CD25^+^ CD69^+^ activated cells) to determine the percentages of CD8^+^ Vα2^+^ T cells that upregulate activation markers.

### Flow cytometry and statistical analysis.

Flow cytometry data acquisition was performed with an LSR-II flow cytometer (BD Biosciences). Data were analyzed as described above using FlowJo 8.8.4 software (TreeStar, Ashland, OR). Statistical analyses were conducted using GraphPad Prism 7 software. The specific test for each analysis is listed in the figure legends. In most cases, an ordinary two-way analysis of variance (ANOVA) was used, followed by *post hoc* analysis with Sidak’s multiple-comparison test (when a set of means was selected to compare) or Tukey’s test (where there was an unequal sample size or the means were compared against every other mean). For pairwise comparisons, an unpaired *t* test with Welch’s correction was used. Differences between groups was considered significant when *P* < 0.05.
